# Phase I and pharmacological trial of lapatinib in combination with gemcitabine in patients with advanced breast cancer

**DOI:** 10.1007/s10637-015-0281-z

**Published:** 2015-09-11

**Authors:** R. van der Noll, W. M. Smit, A. N. M. Wymenga, D. S. Boss, M. Grob, A. D. R. Huitema, H. Rosing, M. M. Tibben, M. Keessen, H. Rehorst, J. H. Beijnen, J. H. M. Schellens

**Affiliations:** Department of Clinical Pharmacology, The Netherlands Cancer Institute, Plesmanlaan 121, 1066 CX Amsterdam, The Netherlands; Department of Internal Medicine, Medisch Spectrum Twente, P.O. Box 50 000, 7500 KA Enschede, The Netherlands; Department of Pharmacy & Pharmacology, The Netherlands Cancer Institute, Plesmanlaan 121, 1066 CX Amsterdam, The Netherlands; Utrecht Institute of Pharmaceutical Sciences (UIPS), David de Wied building Universiteitsweg 99, 3584 CG Utrecht, The Netherlands

**Keywords:** Lapatinib, Gemcitabine, Advanced breast cancer, Phase I trial

## Abstract

*Background* Lapatinib has proven efficacy as monotherapy and in combination with capecitabine in patients with metastatic breast cancer (MBC) overexpressing HER2 and/or EGFR. Gemcitabine also has anti-tumor activity in MBC and a favourable toxicity profile. In this phase I study lapatinib and gemcitabine were combined. *Methods* Female patients with advanced BC were given lapatinib once daily (QD) in 28-day cycles with gemcitabine administered on day 1, 8 and 15. Physical examinations, vital signs and blood sampling for hematology, clinical chemistry and pharmacokinetics (PK) and radiological assessments of disease were performed at regular intervals. *Results* In total, 33 patients were included. Six dose-limiting toxicities were observed, mostly grade 3 increases in liver function tests. Most common toxicities were fatigue (73 %), nausea (70 %), diarrhea (58 %), increases in ALAT and ASAT (55 and 52 %, respectively) and rash (46 %). The maximum tolerated dose was lapatinib 1250 mg QD with gemcitabine 1000 mg/m^2^. Lapatinib and gemcitabine PK did not appear to be influenced by each other. Anti-tumor activity was observed with one patient (4 %) showing complete response and six (23 %) partial response. *Conclusion* Despite a slightly increased toxicity profile compared to their respective monotherapies, lapatinib and gemcitabine can be safely combined while showing signs of anti-tumor activity.

## Introduction

Globally, breast cancer is the most frequently diagnosed cancer and the leading cause of cancer death in both economically developed and developing countries. It accounts for 23 % of new cancer cases and 14 % of cancer deaths worldwide (data from 2008) [1].

In the 1980s, it was discovered that overexpression of human epidermal growth factor receptor 2 (HER2/neu) is associated with poor patient prognosis and survival [2]. HER2 and its rodent analogue neu are oncogenic receptors that, when activated, can trigger multiple intracellular signalling pathways, such as the mitogen-activated protein kinase (MAPK)- and phosphoinositide 3-kinase (PI3K)-pathway, leading to cell survival and proliferation [3, 4]. Therefore, this receptor rapidly became an attractive target for inhibition, leading to the development of trastuzumab (Herceptin®) [5]. This humanized, monoclonal antibody against HER2/neu has since then become an effective treatment option for women with metastatic, HER2-overexpressing breast cancers, both as monotherapy and in combination with chemotherapy [6]. It has also shown to improve survival in the adjuvant setting in patients with HER2-overexpressing breast cancer [7].

Unfortunately, the majority of the patients treated with trastuzumab tend to develop resistance to this drug within 1 year after starting treatment. Anti-tumor activity may be regained by switching the combination of trastuzumab and chemotherapy into trastuzumab plus another cytotoxic agent. However, development of new therapeutic strategies is needed to overcome trastuzumab resistance [8]. Lapatinib is a dual tyrosine kinase inhibitor, targeted against both the epidermal growth factor receptor (EGFR) and HER2/neu [9]. It has shown moderate clinical anti-tumor activity as monotherapy in patients overexpressing HER2/neu and/or EGFR and has a relatively mild toxicity profile with main adverse events consisting of diarrhea and rash [10–12]. Importantly, it has shown anti-tumor activity in patients that had previously progressed on trastuzumab [13]. Adding lapatinib to a chemotherapy regimen has proven to increase its anti-tumor activity, as evidenced by the FDA and EMA approval of lapatinib in combination with capecitabine for previously treated, metastatic, HER2-overexpressing breast cancer [14, 15].

Toxicities observed with this combination in the phase III and registration study only showed increased diarrhea and rash when compared to capecitabine alone, as was expected [16]. These encouraging results warrant further research into combining lapatinib with other cytotoxic agents.

Gemcitabine is a prodrug, which is intracellularly metabolized to its active forms difluorodeoxycytidine diphosphate and triphosphate (dFdCDP and dFdCTP). dFdCDP reduces the number of natural deoxynucleotides, including deoxycytidine triphosphate (dCTP). Additionally, dFdCTP competes with dCTP for incorporation into the DNA and subsequently inhibits DNA synthesis, resulting in cell death [17]. The first approval for gemcitabine was as a treatment modality for pancreatic cancer [18–20]. Due to its favourable toxicity profile, gemcitabine can be combined with other anti-cancer treatments, which for instance led to its use in patients with non-small cell lung cancer (NSCLC) and bladder cancer in combination with cisplatin [21, 22].

Since gemcitabine has shown activity in patients with metastatic breast cancer [23, 24], this study intended to investigate whether a combination of lapatinib and gemcitabine could be a feasible treatment option for women with advanced breast cancer. In order to explore the anti-tumor activity of this combination the optimal dose and schedule had to be determined.

## Patients and methods

All patients provided written informed consent. The study was conducted in accordance with the Declaration of Helsinki, Good Clinical Practice and applicable regulatory requirements. The study was approved by the local ethical review board.

### Patient selection and eligibility

This study was performed at the Netherlands Cancer Institute (NKI) in Amsterdam, the Netherlands and Medisch Spectrum Twente (MST) in Enschede, the Netherlands. It was conducted in women with advanced breast cancer who had previously been treated with an anthracycline and a taxane (either in adjuvant or palliative setting) and were then considered to be candidates for palliative chemotherapy with gemcitabine. Eligibility criteria included: age ≥ 18 years; ECOG performance status ≤ 2; measurable diseases according to Response Evaluation Criteria in Solid Tumors (RECIST); adequate bone marrow, hepatic and renal function (as evidenced by thrombocytes ≥ 100*10^9^/L; absolute neutrophile count ≥ 1.5*10^9^/L; hemoglobin ≥ 6.2 mmol/L; total bilirubin ≤ 1.5 × upper normal limit (ULN); serum aspartate aminotransferase (ASAT) and alanine aminotransferase (ALAT) ≤ 2.5 × ULN and creatinine ≤ 1.5 × ULN); previously treated with trastuzumab (in case of HER2-overexpression); no other investigational drugs within 30 days prior to start. Furthermore, no concomitant medication classified as a CYP3A4 inducer or inhibitor was allowed, since lapatinib is a CYP3A4 substrate. Patients with a clinically significant cardiac impairment (left ventricular ejection fraction (LVEF) of ≤ 50 %) or unstable ischemic heart disease including a myocardial infarction (<3 months of study entry) were not included in this study.

### Study design and procedures

The primary objectives of this phase I study were to determine the safety and tolerability, maximum tolerated dose (MTD), dose-limiting toxicities (DLTs) and optimal treatment schedule of lapatinib combined with gemcitabine. Secondary objectives were to explore the pharmacokinetic (PK) profile and anti-tumor activity of the combination.

Dose escalation in this study followed a classical 3 + 3 design. DLTs were defined as any of the following events determined to be possibly, probably or definitely related to treatment during the first cycle: grade ≥ 3 non-hematological toxicities; grade ≥ 3 nausea, vomiting or diarrhea in the presence of maximal support; grade 4 thrombocytopenia; grade 4 neutropenia for > 7 consecutive days; or grade 3 febrile neutropenia.

The cohort below the non-tolerated dose (i.e., the cohort in which 2 or more out of 6 patients experiences a DLT) was expanded with 6 patients to confirm this dose-level as the MTD.

### Drug administration and dosing schedule

One treatment cycle was defined as 28 days. Lapatinib was administered orally daily in escalating doses, starting from 750 mg once daily (QD). Gemcitabine was given as a standard 30-min intravenous infusion on day 1, 8 and 15 of every cycle.

### Safety evaluations

Physical examinations and the assessments of vital signs, performance status and routine clinical chemistry and hematology were performed at baseline and on day 1, 8 and 15 of the first 2 treatment cycles and on day 1 and 8 of subsequent cycles. A 12-lead ECG was performed at baseline and at the start of every treatment cycle, starting at cycle 2. A chest X-ray and MUGA-scan for measurement of the LVEF was made at baseline and after every 2 treatment cycles.

Adverse events were recorded at every visit and graded according to the National Cancer Institute Common Terminology Criteria of Adverse Events (NCI-CTCAE) version 3.0 [25].

### Pharmacokinetic and pharmacodynamic analysis

Plasma samples for the bioanalysis of lapatinib were collected at predose and 30 min, 1, 2, 4, 8 and 24 h after oral administration. Additional plasma samples were taken predose on day 8, 15 and 29 (cycle 2 day 1). Plasma concentrations of lapatinib were measured using a validated liquid chromatography/tandem mass spectrometry method [26].

Blood samples for gemcitabine were collected prior to, at the end of infusion and 1, 2, 4, 8 and 24 h after start of infusion. Plasma gemcitabine (2′,2′-difluorodeoxycytidine, dFdC) and its metabolite 2′,2′-difluorodeoxyuridine, dFdU) concentrations were analyzed as described before [27], with the exception that tetrahydrouridine was not added to the sodium-heparine blood collection tubes. Additionally, intracellular concentrations of the active metabolite of gemcitabine, difluorodeoxycytidine triphosphate (dFdCTP), were measured as described previously in peripheral blood mononuclear cells (PBMCs) [28] prior to, at the end of infusion and 2 and 24 h after start of infusion.

### Assessment of anti-tumor activity

Tumor measurements were performed by CT or MRI-scan at baseline and after every 2 treatment cycles. Scans were then evaluated according to Response Evaluation Criteria in Solid Tumors (RECIST) version 1.0 [29].

Furthermore, the tumor markers cancer antigen 15.3 (CA15.3) and carcinoembryonic antigen (CEA) were measured at baseline and after every 2 treatment cycles.

## Results

### Patient characteristics

In total, 33 female patients with advanced breast cancer were included in this study, between November 2007 and November 2012. Table 1 shows the patient characteristics.

**Table 1:** Patient characteristics

Characteristic		
No. of patients		33
Age, years	Median (range)	50 (36–73)
Ethnic origin, n (%)	Caucasian	30 (91)
	East/Southeast Asian	2 (6)
	Hispanic	1 (3)
ECOG performance status, n (%)	0	11 (33)
	1	18 (55)
	2	4 (12)
No. of prior chemotherapy, n (%)	2	6 (18)
	3	9 (27)
	4	11 (33)
	≥5	7 (21)
Prior hormonal therapy, n (%)		23 (70)
Prior immunotherapy, n (%)		21 (64)
Prior radiotherapy, n (%)		29 (88)
HER2-overexpression, n (%)	Yes	22 (67)
	No	10 (30)
	Unknown	1 (3)
LVEF, %	Median (range)	56 (52–73)
CA15.3, kU/L	Median (range)	110 (11–770)

Patient characteristics. *LVEF* left ventricular ejection fraction, *CA* cancer antigen

Four patients had to be replaced per protocol because they received < 80 % of study medication during the first cycle to ensure that at least 3 subjects were evaluable for safety over the whole course of the first cycle. The median age of patients in this study was 50 years (36–73). They were heavily pre-treated with approximately half of all patients (54 %) having received ≥ 4 lines of chemotherapy. Additionally, 70 % of the patients received prior hormonal therapy, 64 % were previously treated with trastuzumab and 88 % had received prior radiotherapy. HER2-overexpression was demonstrated in the tumors of 22 patients (67 %).

### Dose-Limiting Toxicities (DLTs) and Maximum Tolerated Dose (MTD)

Five escalating dose-levels were explored in this study (see table 2). In total, six DLTs were observed across four dose-levels. In five cases, the DLT concerned a grade 3 increase in ASAT and/or ALAT. This increase generally occurred 1 to 2 weeks after start of the first cycle, leading to an interruption of lapatinib dosing and omission of gemcitabine infusions. Two patients showed progression of disease after this DLT and did not re-start treatment; in the other three patients the liver function tests recovered and cycle 2 was administered, albeit with a dose reduction of gemcitabine with 25 % and lapatinib with 250 mg. Additionally, one DLT of grade 3 diarrhea was observed at the highest dose-level explored, despite maximal support with loperamide and ciprofloxacin. Lapatinib was interrupted and gemcitabine omitted. This patient was not re-started on treatment.

**Table 2:** Dose-levels

Dose-level	Lapatinib QD (mg)	Gemcitabine (mg/m2)
1	750	750
2	750	1000
3	1000	1000
4	1250	1000
5	1500	1000

The dose-levels explored in this study

At the time the study was ongoing, only one DLT (grade 3 elevation in both ASAT and ALAT) was recorded in dose-level 3, which was then expanded. Since no other DLTs were seen in the dose expansion, the dose was escalated to dose-level 4. A second DLT (grade 3 elevation in ALAT) in dose-level 3 was not noted until study data were carefully re-analysed.

Since dose-level 5 was not considered to be tolerable with two out of six patients experiencing a DLT, the MTD was set at dose-level 4, consisting of lapatinib 1250 mg QD (day 1–28) and gemcitabine 1000 mg/m^2^ (day 1, 8 and 15). This dose-level was then expanded with six additional patients, none of whom experienced DLTs.

### Adverse events

Treatment-related adverse events (TRAEs) observed in ≥ 10 % of patients in this study are presented in Table 3.

**Table 3:** Treatment-related adverse events

Adverse event		Dose-level					Total
		1	2	3	7	5	
	No. of patients	3	4	8	10	8	33
Fatigue	All grades	2 (67)	3 (75)	7 (88)	7 (70)	5 (63)	24 (73)
	Grade ≥3	0	0	1 (13)	0	0	1 (3)
Nausea	All grades	1 (33)	4 (100)	7 (88)	4 (40)	7 (88)	23 (70)
	Grade ≥3	0	0	1 (13)	0	0	1 (3)
Diarrhea	All grades	2 (67)	2 (50)	4 (50)	5 (50)	6 (75)	19 (58)
	Grade ≥3	0	0	1 (13)	1 (10)	1 (13)	3 (9)
Elevated ALAT	All grades	1 (33)	3 (75)	5 (63)	5 (50)	4 (50)	18 (55)
	Grade ≥3	0	1 (25)	2 (25)	1 (10)	2 (25)	6 (18)
Elevated ASAT	All grades	1 (33)	3 (75)	5 (63)	4 (40)	4 (50)	17 (52)
	Grade ≥3	0	1 (25)	3 (38)		1 (13)	5 (15)
Rash*	All grades	0	2 (50)	4 (50)	5 (50)	4 (50)	15 (46)
	Grade ≥3	0	0	0	0	0	0
Neutropenia	All grades	2 (67)	2 (50)	1 (13)	5 (50)	3 (38)	13 (39)
	Grade ≥3	2 (67)	2 (50)	1 (13)	5 (50)	3 (38)	13 (39)
Vomiting	All grades	0	2 (50)	5 (63)	2 (20)	2 (25)	11 (33)
	Grade ≥3	0	0	1 (13)	0	0	1 (3)
Fever	All grades	1 (33)	1 (25)	2 (25)	2 (20)	2 (25)	8 (24)
	Grade ≥3	0	0	0	0	0	0
Mucositis#	All grades	0	1 (25)	1 (13)	2 (20)	3 (38)	7 (21)
	Grade ≥3	0	0	0	0	0	0

Treatment-related adverse events per dose-level. *includes desquamation, acneiform, erythema. # both clinical examination and symptomatic

The most commonly observed possibly, probably or definitely TRAEs were fatigue (73 %), nausea (70 %), diarrhea (58 %), increases in ALAT and ASAT (55 and 52 %, respectively), rash (46 %) and neutropenia (39 %).

Adverse events that were grade 3 or higher mostly consisted of neutropenia (39 %), elevated ALAT and ASAT (18 and 15 %, respectively) and diarrhea (9 %).

Interrupting lapatinib dosing and omitting gemcitabine infusion improved the observed toxicities and led to recovery of hematological and biochemical laboratory values. Nausea and diarrhea were mostly mild (grade 1–2), but in some cases these toxicities required concomitant treatment. Nausea was initially treated with metoclopramide, however, in more severe cases granisetron was required. Diarrhea sometimes required treatment with loperamide. Rash was mild (grade 2 or lower) in all cases and responded to local treatment, such as with metronidazole crème or systemic anti-histaminic treatment, such as with levocetirizine.

While there were five patients who demonstrated a median decrease in LVEF of 10 % (range 8–14 %) after two cycles, the patients who remained on study for six cycles or longer showed stable LVEF values.

### Pharmacokinetics

**Fig. 1:**
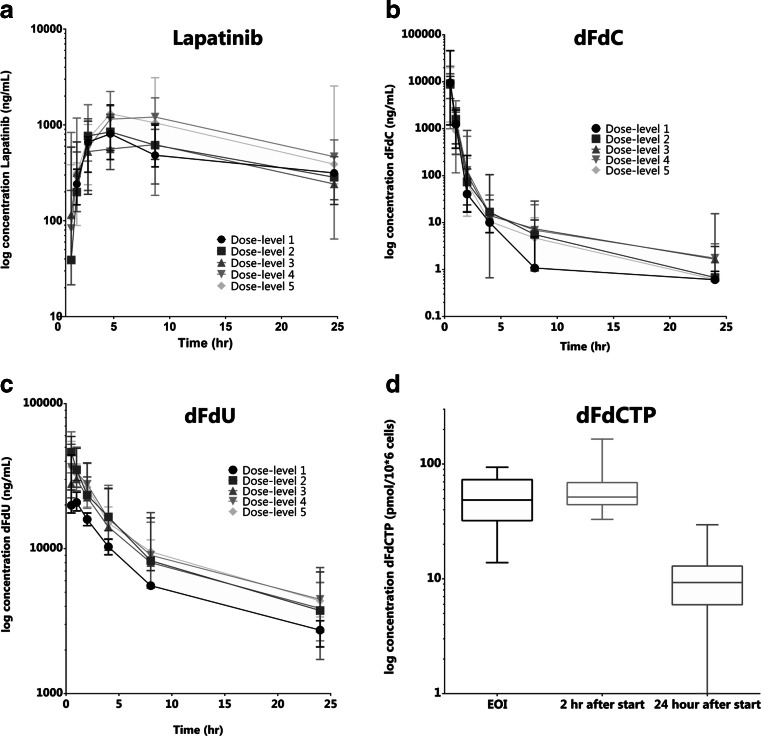
Plasma concentration-time curves of lapatinib (**a**), dFdC (**b**), dFdU (**c**) and a boxplot showing the median and range of dFdCTP over time (**d**)

PK parameters and plasma concentration-time curves of lapatinib and gemcitabine (dFdC and dFdU) and intracellular concentrations of dFdCTP are presented in table 4 and Fig. 1.

**Table 4:** Pharmacokinetic parameters

Dose-level	1	2	3	4	5
Lapatinib QD (mg)	750	750	1000	1250	1500
Gemcitabine (mg/m2)	750	1000	1000	1000	1000
Lapatinib (median (range))					
	*n* = 3	*n* = 4	*n* = 7	*n* = 9	*n* = 7
Cmax (ng/mL)	807 (436–1614)	872 (540–1200)	818 (450–1294)	1258 (573–2231)	1314 (1030–3108)
Dose-normalized Cmax (ng/mL*mg)	1.1 (0.6–2.2)	1.2 (0.7–1.6)	0.8 (0.5–1.3)	1.0 (0.5–1.8)	0.9 (0.7–2.1)
Tmax (hr)	4.0 (4.0–4.1)	4.0 (2.1–4.4)	4.0 (2.0–8.1)	4.0 (2.1–8.0)	4.2 (4.0–6.9)
Gemcitabine (median (range))					
dFdC	*n* = 3	*n* = 4	*n* = 7	*n* = 9	*n* = 7
Cmax (ug/mL)	9.5 (1.2–4.6)	9.1 (7.4–13.0)	10.9 (4.4–14.8)	9.2 (5.0–21.5)	10.2 (1.4–20.0)
Dose-normalized Cmax (ng/mL*mg)	7.6 (1.0–38.4)	5.4 (4.7–6.2)	6.2 (2.3–7.4)	4.8 (0.6–12.1)	5.7 (0.9–10.5)
AUC0-24 (ug*hr/mL)	7.1 (1.0–24.8)	6.4 (5.2–7.6)	7.9 (3.3–11.3)	7.8 (0.6–17.1)	6.8 (1.2–10.5)
dFdU	*n* = 3	*n* = 4	*n* = 7	*n* = 9	*n* = 7
Cmax (ug/mL)	20.8 (18.2–44.3)	46.4 (29.5–59.3)	33.5 (26.4–52.4)	36.4 (33.3–63.9)	37.6 (26.8–54.9)
Dose-normalized Cmax (ng/mL*mg)	16.6 (14.3–36.9)	24.1 (18.4–37.8)	19.9 (16.5–26.2)	21.4 (17.9–39.9)	21.4 (15.8–28.9)
AUC0-∞ (ug*hr/mL)	196 (185–202)	282 (232–505)	272 (188–496)	306 (226–461)	324 (272–359)
Terminal half-life (t1/2) (hr)	9.1 (8.1–11.5)	9.0 (7.5–9.9)	10.3 (6.3–11.0)	8.9 (8.0–9.7)	9.6 (8.1–13.4)
dFdCTP	*n* = 3	*n* = 3	*n* = 5	*n* = 2	NA
Cmax (pmol/10*6 cells)	63 (51–69)	59 (54–62)	42 (33–84)	147 (128–165)	NA
Tmax (hr)	2.0 (0.6–2.0)	0.8 (0.6–2.0)	1.9 (0.7–2.1)	2.0 (2.0–2.1)	NA

Pharmacokinetic parameters of lapatinib and gemcitabine (dFdC, dFdU and dFdCTP) per dose-level

#### Lapatinib

Plasma samples for the PK of lapatinib were collected and analyzed in 28 patients. The PK of lapatinib showed high interpatient variability (see Fig. 1a). The median time to maximum concentration (T_max_) was mostly observed at 4.0 h (range 2.0–8.1). The maximum plasma concentration (C_max_) increased with dose and this increase appeared to be dose-proportional. Since the blood sampling did not cover > 80 % of the area under the plasma concentration-time curve (AUC) due to a long elimination phase, the AUC_0-∞_ and terminal half-life (t_1/2_) could not be determined from the obtained data.

### Gemcitabine

The PK parameters for dFdC and dFdU were determined in plasma collected from 30 patients. See Fig. 1b and c.

The dose-normalized C_max_ and AUC_0–24_ did not differ significantly amongst dose-levels, although there was high variation in the first three patients on study.

For the metabolite dFdU, the C_max_ followed closely after dFdC, indicating rapid formation of this metabolite. Both C_max_ and AUC_0-∞_ increased dose-proportionally across the different dose-levels.

### Pharmacodynamics

PBMCs of 26 patients were available for the analysis of intracellular dFdCTP concentration. However, in about half of the patients the dFdCTP measurements were below the lower limit of quantification (LLOQ) and were therefore not used in further analyses. The results obtained in 13 patients are shown in Fig. 1d. High variability was observed; therefore AUC_0-∞_ and t_1/2_ could not be determined.

### Anti-tumor activity

Out of the 33 patients included in this study, 26 had at least one response evaluation (see table 5); seven patients had gone off study without receiving one full cycle because of rapid clinical disease progression. One patient (4 %) demonstrated radiological complete response (CR) and another six patients (23 %) achieved a partial response (PR), resulting in an overall response rate (ORR: CR + PR) of 27 %. Five of the patients that had radiological PR also demonstrated clear reductions in their tumor markers (both CA15.3 and CEA). An additional 12 patients (46 %) had stable disease (SD) as best response. Radiological responses were mostly observed at higher dose-levels (≥ level 3). Furthermore, the patient with CR and 5/6 patients with PR demonstrated tumors that overexpressed HER2.

**Table 5:** Anti-tumor activity

Dose-level	No. of patients	Median treatment duration (weeks), range	Response by RECIST, n (%)				
			*CR*	*PR*	*SD*	*PD*	*NE*
1	3	14 (2–30)	0	0	1 (33)	2 (67)	*0*
2	4	8.5 (7–14)	0	0	4 (100)	0	0
3	8	14 (0–32)	1 (12)	1 (12)	2 (25)	2 (25)	1 (12)
4	10	6 (0–39)	0	3 (30)	3 (30)	2 (20)	1 (10)
5	8	10.5 (1–49)	0	2 (25)	2 (25)	1 (12)	1 (12)
Total	33	8 (0–49)	1 (3)	6 (18)	12 (36)	7 (21)	3 (9)

Best tumor response by RECIST observed during this study per dose-level. *CR* complete response, *PR* partial response, *SD* stable disease, *PD* progressive disease, *NE* not evaluable.

Median duration on study for the evaluable population was 13 weeks, ranging from 2 to 49 weeks.

## Discussion

In this phase I study, the combination of lapatinib with gemcitabine did not result in unexpected toxicities. The MTD of the combination was defined at lapatinib 1250 mg QD (day 1–28) with gemcitabine 1000 mg/m^2^ (day 1, 8 and 15). The most frequently observed TRAEs were in line with toxicities that are observed with gemcitabine and lapatinib monotherapy. Both frequency and severity of TRAEs increased with escalating dose-levels. The DLTs that were observed in this study did not correspond to higher exposures to study drug, except for the patient who experienced grade 3 diarrhea despite maximal support with loperamide and ciprofloxacin. This patient showed the highest exposure to lapatinib out of all patients on study, which could explain the persisting toxicity. The most common adverse events seen with lapatinib, diarrhea and rash (42 and 31 %, respectively, in the lapatinib monotherapy phase I study) [10], were also observed in this study albeit with slightly increased frequency (58 and 46 %, respectively). In all cases, rash was mild (grade 1–2) and appeared to respond to local or systemic treatment. The diarrhea was also mostly mild and manageable with concomitant treatment of loperamide.

Hematological and biochemical toxicities, such as neutropenia and elevated ALAT and ASAT, were also increased in the lapatinib/gemcitabine combination (39, 55 and 52 %, respectively) when compared to gemcitabine monotherapy (26, 39 and 31 %, respectively) [18].

Overall, these data suggest that combining these compounds does result in slightly increased toxicity when compared to either of these agents alone. This resulted in a lower lapatinib dose in the MTD of this study (1250 mg) than the dose that is recommended as monotherapy (1500 mg). Gemcitabine however could be administered at the recommended monotherapy dose of 1000 mg/m^2^.

The PK of lapatinib showed high interpatient variability. The dose-normalized C_max_ did not differ significantly between the different dose-levels. However, no sampling was done for lapatinib alone thus no direct treatment comparisons could be made. Furthermore, patient numbers were small, making it difficult to draw definitive conclusions about the influence of gemcitabine on lapatinib PK.

Less variation was seen in the PK of gemcitabine (dFdC) and its metabolite dFdU. For both dFdC and dFdU, PK appeared to be similar to gemcitabine monotherapy [30], suggesting that co-administration of lapatinib did not influence gemcitabine PK.

The measurement of dFdCTP was performed in about half of the patients, since many of the samples were below the LLOQ. We suspect that this might be due to the sample processing. Most of the later samples were not washed with ice-cold PBS as described in the validated method [28] or processed on ice, which could have led to an increase in the rate of degradation of dFdCTP. In the subjects where dFdCTP could be determined it showed very high variability, although C_max_ values found were similar to those in literature with similar doses of gemcitabine [31].

Although not the primary objective of this study, the ORR observed in this study (27 % for all evaluable patients; 21 % for evaluable patients whose tumor overexpressed HER2) is in line with the lapatinib/capecitabine (1250 mg continuously/2000 mg/m^2^ on day 1–14) combination (ORR of 22 %) in the phase III trial in a similar population.(16) However, this conclusion has to be considered carefully since patient numbers were much smaller in this phase I study (26 patients evaluable for response compared to 163 patients in the Geyer study) and in this study HER2-expression was not an inclusion criterium.

The combination of lapatinib with gemcitabine (and oxaliplatin) has been investigated clinically before in patients with pancreatic and biliary cancer. In a phase I study in this population the MTD of lapatinib with gemcitabine was set at 1500 mg and 1000 mg/m^2^ respectively, with only 1 DLT of grade 3 diarrhea [32]. A subsequent phase II study of lapatinib/gemcitabine was performed in patients with metastatic pancreatic cancer. Toxicities were mostly hematologic (30 % experienced grade 3 or 4 neutro- or thrombocytopenia); fatigue, diarrhea, elevated ASAT/ALAT and anorexia were the most frequent non-hematological grade ≥ 2 adverse events. Unfortunately, the combination failed to improve the overall survival rate in this patient population [33]. In a recent preclinical study lapatinib was tested in combination with gemcitabine to investigate whether these drugs showed synergistic or antagonistic effects in pancreatic cancer cell lines. There was no clear effect observed and thus the authors concluded that lapatinib may not enhance the anti-tumor effects of gemcitabine in pancreatic cancer [34]. In contrast and consistent with our study, encouraging evidence of lapatinib in combination with gemcitabine was demonstrated in a case report of a female with metastatic, HER2-overexpressing breast cancer who developed a complete clinical response for 1 year after being treated with this combination (lapatinib 1250 mg, later reduced to 1000 mg and gemcitabine 1000 mg/m^2^) [35].

Overall, despite a slight increase in toxicity profile compared to their monotherapies, the combination of lapatinib with gemcitabine appears to be a safe treatment regimen for patients with advanced breast cancer, while showing preliminary signs of anti-tumor activity. Combining these two compounds did not appear to influence each other’s PK profile. Although further studies are needed to confirm that the anti-tumor activity is comparable to the lapatinib/capecitabine combination, the lapatinib combination with gemcitabine could be a new treatment modality for these patients after progressing on treatment with an anthracycline and trastuzumab.
